# Radiologic Evaluation of Odontogenic Sinusitis and Its Etiologic Factors: Lessons Learned from a Retrospective Study with a Proposed Imaging-Guided Management Pathway

**DOI:** 10.3390/jcm15103724

**Published:** 2026-05-12

**Authors:** Kamil Nelke, Monika Morawska-Kochman, Maciej Janeczek, Agata Małyszek, Ömer Uranbey, Klaudiusz Łuczak, Jan Nienartowicz, India Maag, Angela Rosa Caso, Maciej Dobrzyński

**Affiliations:** 1 Maxillo-Facial Surgery Ward, EMC Hospital, Pilczycka 144, 54-144 Wrocław, Poland; 2 Academy of Applied Sciences, Health Department, Academy of Silesius in Wałbrzych, Zamkowa 4, 58-300 Wałbrzych, Poland; 3 Department of Head and Neck Surgery, Otolaryngology, Wrocław Medical University, Borowska 213, 50-556 Wrocław, Poland; 4 Department of Biostructure and Animal Physiology, Wrocław University of Environmental and Life Sciences, Kożuchowska 1, 51-631 Wrocław, Poland; maciej.janeczek@upwr.edu.pl (M.J.); agata.malyszek@upwr.edu.pl (A.M.); 5 Department of Oral and Maxillofacial Surgery, Faculty of Dentistry, Aydın Adnan Menderes University, 09010 Aydın, Türkiye; eomeruranbey@gmail.com; 6 Maxillo-Facial Surgery Ward, Wrocław Medical University, Borowska 213, 50-556 Wrocław, Poland; 7 Private Dental Office, Pawia 56 Street, 54-144 Wrocław, Poland; 8 Department of Oral and Maxillofacial Surgery, University of Siena, Viale Aldo Moro, 2-Siena SI, 53100 Siena, Italy; 9 Department of Pediatric Dentistry and Preclinical Dentistry, Wrocław Medical University, Krakowska 26, 50-425 Wrocław, Poland

**Keywords:** maxillary sinus, odontogenic infection, sinusitis, maxillary sinusitis, tooth-related infection

## Abstract

**Introduction:** Odontogenic sinusitis (ODS) is an underrecognized cause of maxillary sinus inflammation and is frequently associated with dental, periodontal, endodontic, and iatrogenic factors. Accurate identification of the odontogenic source is essential for appropriate treatment planning. Cone-beam computed tomography (CBCT) allows detailed evaluation of the maxillary sinus, adjacent teeth, alveolar bone, and periodontal structures, and may improve the radiologic differentiation of ODS. **Materials and Methods:** This retrospective observational study analyzed radiologic data from patients evaluated and treated by the authors for suspected odontogenic sinusitis between 2019 and 2026. The final study group included 85 patients with CBCT-based evidence of odontogenic pathology affecting the maxillary sinus. CBCT scans were reviewed to identify tooth-related and treatment-related etiologic factors associated with ODS. Based on the radiologic findings, the authors developed a CBCT-based classification of odontogenic etiologies and proposed an imaging-guided management algorithm. **Results:** CBCT identified a broad spectrum of odontogenic factors associated with maxillary sinus disease. The most relevant radiologic patterns included endodontic and periapical pathology, periodontal or combined endo-periodontal disease, post-extraction inflammatory changes, odontogenic cysts, oro-antral communication or fistula, retained roots or teeth, displaced endodontic materials, and grafting or implant-related complications. These findings were organized into 16 radiologic categories reflecting the principal etiologic pathways of ODS. The proposed classification facilitated correlation between radiologic presentation and the recommended dental, surgical, and otolaryngologic treatment approach. **Conclusions:** CBCT is a valuable imaging modality for identifying odontogenic causes of maxillary sinus inflammation and provides more precise diagnostic information than conventional radiography alone. A structured CBCT-based evaluation may improve etiologic diagnosis, support multidisciplinary decision-making, and help guide individualized management of patients with ODS.

## 1. Introduction

Odontogenic sinusitis (OS, ODS) is an important and often underrecognized subtype of maxillary sinusitis that arises from dental infection, dentoalveolar pathology, or iatrogenic dental causes. It most commonly presents as unilateral maxillary sinus disease, although more extensive sinonasal involvement and, in rare cases, extra-sinus complications may occur. The influence of teeth on sinus condition and the occurrence of maxillary sinusitis is still underestimated and undervalued in many cases. In each case of odontogenic sinusitis, not only the dentist, oral surgeon, maxillo-facial surgeon, and otolaryngologist (ORL), but also the radiologist has a very important role in the treatment and diagnostics of the following inflammatory process. Many review papers nowadays indicate that ODS requires multidisciplinary treatment, and only the combined team approach might grant more successful final results after treatment [1,2].

Cases of rhinosinusitis (RHS) have different treatment outcomes. Usually, careful assessment of the patient’s dental status and CBCT findings is essential to confirm or exclude a potential odontogenic origin, which has been reported in 25–55% of rhinosinusitis cases. Some other basic radiological data can be useful in initial diagnostics; however, only a 3D imaging modality like CT or CBCT is sufficient in ODS. In routine cases, otolaryngological examination includes nasal endoscopy to assess unilateral middle meatal purulence, osteomeatal complex (OMC) patency, and the presence of additional findings such as foul odor, rhinorrhea, and sinus opacification on CT/CBCT [1,2,3,4,5]. Additionally, the condition of other sinuses, the presence of a deviated nasal septum, enlarged nasal meatus, and inadequate nasal ventilation should also be noted. According to recent studies, ODS most commonly occurs in the fifth decade of life, usually involves the maxillary sinus, tends to present with chronic symptoms, affects both sexes similarly, and is associated with an odontogenic etiologic factor (endodontic, periapical, periodontal, or other dental related) and can also lead to dangerous bone inflammations, orbital involvement, intracranial complications or other serious conditions. This condition is referred to as extra-sinus spread and is particularly concerning in children because of their venous anatomy and bony canal systems, which may facilitate a more rapid extension of the infection. Furthermore, bacteriological profile in ODS has different features than in classic rhinosinusitis [6,7,8]. FESS (functional endoscopic sinus surgery) or ESS (endoscopic sinus surgery), followed by a detailed dental treatment (endodontic, periodontal, dentoalveolar surgery, ex–teeth removal), in some cases even open surgery with classic Caldwell-Luc procedures (antrostomy) are necessary to improve sinus ventilation, remove all inflamed tissues or foreign bodies from the sinus and sometimes even a surgical approach for closure of any oro-antral communication/fistula surgery is mandatory. The usage of antibiotics is case-related, and it is not considered a golden standard; however, if purulent secretions are subjected to microbiological analysis, antibiotic therapy can be tailored accordingly. Secondly, nasal rinses, nasal congestion, and other aerosols/solutions are necessary to improve the ventilation and sinus healing processes. Because ODS is often associated with polymicrobial sinus flora, antibiotic therapy is frequently considered as part of case-specific management. All ODS evaluations should include the description of sinus opacification, the status of OMC, and establish the degree of maxillary sinus mucosal thickening over time [4,5,6,7,8,9,10].

Some studies indicate that CBCT (cone-beam computed tomography) is a very useful and highly effective diagnostic tool for ODS. Tooth-bearing structures and the proximity of teeth to the maxillary sinus, as well as periapical inflammation, sinus floor erosion, mucosal thickening, cyst formation, and other related findings, can be readily assessed. On the contrary, clinical symptoms such as rhinorrhea, cheek pain, foul odor, nasal congestion, postnasal drip, headache, sinus pain, gingival swelling, tooth pain, or similar are present; however, these findings are not specific to ODS and do not by themselves confirm an odontogenic origin. Differentiation between acute or chronic rhinosinusitis and ODS requires detailed imaging, particularly CBCT, together with clinical evaluation to confirm or exclude tooth-related factors [5,6,7,8,9,10,11,12,13,14,15,16,17,18].

Numerous studies worldwide have highlighted the influence of dental factors on maxillary sinus disease [1,12,13,14,15,16,17,18,19,20,21,22,23,24,25,26,27,28,29,30]. Adequate oral hygiene and proper dental status appear to influence maxillary sinus health and the occurrence of odontogenic sinusitis. ODS most commonly presents as a unilateral disease and may be associated with partial or complete sinus opacification on CT. A wide variety of dental, endodontic, osseous, and periodontal factors may contribute to the development of odontogenic sinusitis. Findings visible on conventional sinus CT may differ from those identified on CBCT, which is better suited for the evaluation of the jaw bones, teeth, and periodontal tissues [15,16,17,18,19,20]. The use of CBCT facilitates detailed evaluation of the jaw bones and teeth [1,17,18,19,20,21,31,32,33,34,35,36,37,38,39].

In the present study, the authors propose an evaluation of the etiological factors of odontogenic sinusitis based on treated patient cases and collected radiological data, with special emphasis on the role of CBCT and its correlation with treatment algorithms used by the authors’ multidisciplinary team.

## 2. Materials and Methods

### 2.1. CBCT Study Characteristics

A total of 120 radiological examinations showing different types of maxillary sinusitis and related lesions were initially reviewed. All evaluated data were derived from patients who had been treated, diagnosed, or consulted by the Authors’ team. Among these 120 radiological images, potential sources of odontogenic sinusitis (ODS) were assessed; however, only 85 cases with CBCT examinations and radiological findings consistent with ODS were ultimately included in the study.

This study was designed as a retrospective observational radiological analysis based on cases collected from the authors’ retrospective clinical database between 2019 and 2026. The unit of analysis was the individual patient case with available CBCT imaging and radiological features consistent with ODS. The study focused specifically on cases of odontogenic sinusitis, rather than on a mixed cohort of all maxillary sinus pathologies.

All included examinations were CBCT scans obtained with various fields of view (FOVs) and imaging protocols. Although the scans were acquired using different CBCT protocols and FOVs, all included examinations allowed assessment of the maxillary dentition, adjacent alveolar bone, and the affected maxillary sinus. Cases in which these structures were not sufficiently visualized were excluded. The purpose of the radiological analysis was to identify the likely odontogenic source of sinus disease and to support a practical imaging-based categorization of ODS-related etiological factors and their possible therapeutic implications.

All CBCT examinations included in the study belonged to patients who had been treated, consulted, or operated on by the authors in relation to odontogenic disease affecting the maxillary sinus. Institutional review board approval was obtained for the study under the following reference number: 2-4/BNR-2022.

The authors assessed the status of the maxillary sinus and adjacent dentoalveolar structures primarily on CBCT imaging. Particular attention was paid to the condition of the teeth, periodontal tissues, alveolar bone, and other coexisting radiological findings that could indicate an odontogenic source of sinus disease. The primary analysis focused on radiological features visible on CBCT and used these findings to formulate an imaging-based practical categorization of odontogenic causes of maxillary sinus disease encountered in the present cohort. Additional imaging modalities, when available and when presented in selected figures, were used only for complementary clinical illustration and not as the primary basis for etiological categorization. Patient age, sex, and systemic comorbidities were not treated as primary study variables because the main aim of the paper was to present the radiological identification of etiological factors of odontogenic sinusitis and their possible treatment implications. A close radiological evaluation of teeth-bearing structures and their close proximity is the golden standard for ODS identification, regardless of the voxel size range, machines used and reconstruction parameters, only if entire sinus and surrounding bone is clearly visible in the imaging.

### 2.2. Methods

The authors aimed to propose a method for identifying potential causes of ODS based on detailed radiological evaluation (CBCT, CR, and panoramic radiography). A detailed CBCT evaluation provides most of the information in terms of both maxillary sinuses, bone condition, periodontal status, teeth anatomy, and alveolar bone evaluation, which helped in the identification of tooth-related etiological factors. Based on a review of reported ODS causes, clinical algorithms, and their own experience, the authors propose the most accurate and recommended treatment protocols.

The inclusion criteria were as follows: (i) availability of CBCT and Panoramic scans showing the maxillary sinuses, maxillary bone, adjacent teeth, and periodontal tissues in full; (ii) radiologically confirmed ODS; (iii) presence of clinical symptoms of ODS for more than three months; (iv) patients who were treated, consulted, and operated on by the authors; (v) presence of atypical maxillary sinus symptoms related to dental or tooth-associated factors; (vi) all surgical procedures performed by the two lead surgeons (K.N. and K.Ł.); and (vii) availability of additional radiographic examinations to assess the full extent of the tooth-bearing structures and identify possible causes of ODS.

The exclusion criteria were as follows: (i) CBCT scans that did not fully visualize the teeth, maxillary sinuses, and adjacent bony structures; (ii) CBCT scans showing only one maxillary sinus; (iii) patients in whom otolaryngological treatment alone resulted in improvement of both sinus status and maxillary sinusitis-related symptoms despite radiological findings suggestive of ODS; (iv) patients who were not treated, consulted, or operated on by the authors; (v) patients who were unwilling to undergo further CBCT or Panoramic imaging for ODS diagnosis; (vi) patients who were unwilling to participate in the treatment approach proposed by the authors; and (vii) patients who declined both CBCT evaluation and the scheduled dental procedures.

Statistical analysis focused primarily on quantitative variables, and the chi-squared test and Fisher’s exact test were used for analysis. All pairs of variables were compared, and statistically significant when *p* < 0.05. Statistica 14.0 software was used for measurements. The strength of the association between the analyzed factors was determined using the odds ratio (OR) with 95% confidence interval (95% CI). Results with a *p*-value less than 0.05 (*p* < 0.05) were considered statistically significant.

### 2.3. Tooth-Related Odontogenic Inflammation

The relationship between the teeth, tooth-bearing bony structures, and the development of odontogenic inflammation may be classified into several radiologically identifiable patterns, as outlined in points (A–O). A thorough CBCT assessment should evaluate not only the involved tooth and its internal structure, but also the periodontal tissues, maxillary alveolar bone, alveolar process, alveolar recess of the maxillary sinus, and sinus ventilation. For the purposes of this study, all CBCT examinations were re-evaluated by experienced members of the authors’ team, including M.M.K. and J.N., to identify radiological features potentially associated with odontogenic sinus disease. Based primarily on these CBCT findings, the etiological factors related to ODS in the study cohort were categorized into the following groups, each of which may imply dental, surgical, otolaryngological, or combined treatment. The proposed classification (A–P) is presented in Table 1.

**Table 1:** Authors proposal of ODC classification based on CBCT-radiological/clinical signs and symptoms.

ODS Types and Coding	ODS Description	Figure Ref.	Treatment Ref. Fig.
**A**	Not healed periapical lesions after an endodontic treatment	Figure 1A	[4]
**B**	Insufficient endodontic treatment, leaving a dental canal untreated	Figure 1B	[4]
**C**	Endodontic material or other dental filler pushed through the teeth apex in the maxillary bone or within the sinus antrum—not related to aspergilloma formation	Figure 1C	[5]
**D**	Undiagnosed teeth, dental pulp necrosis, and chronic dental inflammation causing sinus inflammation with or without a dental crown destruction	Figure 1D	[4]
**E**	Endo-perio or perio-endo related inflammation causing a secondary maxillary sinusitis with non-vital pulp in the teeth	Figure 1E	[6]
**F**	After teeth removal, the formation of an inflamed socket, which is mostly not treated in time, causes local inflammation and secondary maxillary sinusitis	Figure 1F	[7]
**G**	The formation and presence of an odontogenic cyst not exceeding the maxillary alveolar process and causing secondary sinusitis	Figure 2A	[7]
**H**	Fully developed odontogenic cyst invading the maxillary atrium and causing bone deformity	Figure 2B	[7]
**I**	A combination of all of the above factors (1–8)	Figure 2C	[7]
**J**	Atypical secondary sinus infection caused by poor periodontal status with a vital tool	Figure 2D	[6]
**K**	Not diagnosed and not properly closed oro-antral communication after a tooth removal	Figure 2E	[7]
**L**	Unerupted or retained teeth in the maxillary sinus, causing a secondary mucous accumulation, cyst formation, and possible symptoms of inflammation when the retained cyst might become inflamed	Figure 2F	[7]
**M**	Retained and forgotten teeth apexes or residual dental parts embedded into the bone close to the floor of the sinus	Figure 3A	[7]
**N**	Endodontic material after dental root treatment, with the formation of an aspergilloma infection around the endodontic material, causing an odontogenic sinusitis	Figure 3B	[7]
**O**	Bone filling material used for socket preservation, bone fillings, sinus lift materials, and other materials that protrude towards the maxillary sinus (xenograft, allograft, autograft)	Figure 3C	[8]
**P**	Unfinished or undiagnosed dental treatment (>6 months after past treatment)	Figure 3D,E	[4]

Abbreviations—ODS-odontogenic sinusitis; CBCT-cone-beam computed tomography imaging; Ref—reference. The simplicity of the following table classification is based on its easiest validation method, without hierarchy of most advanced/less favorable factors based on individual-patient specific situations, and are not overlapping. Authors presented most common factors found based on selected Endodontic, Periodontal, Post-extractive and Iatrogenic factors most commonly established in this paper. Future studies on ODS will take an attempt to standardize the following etiological aspects in more etiological-clinical decision tree made factors.

**Figure 1:**
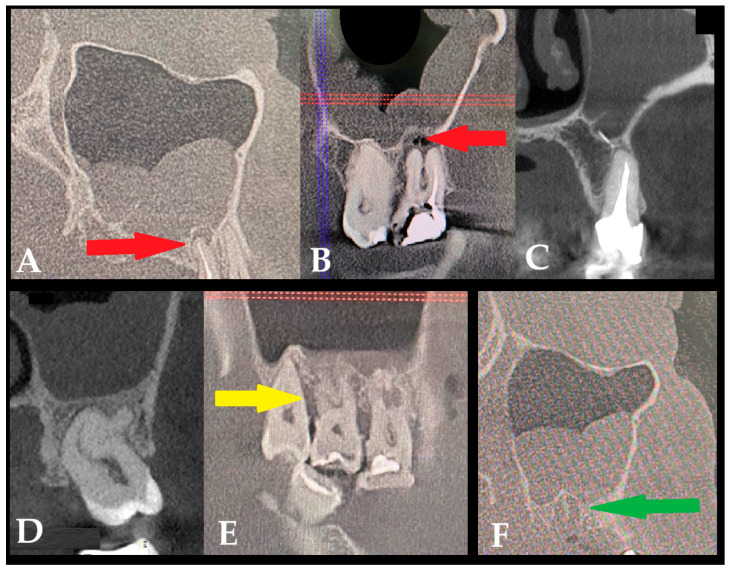
The sources of odontogenic sinusitis (ODS) found in patients’ examination: (**A**) Typical periapical lesion/inflammation that is not healing after an endodontic treatment. (**B**) Inadequate endodontic treatment—note one root canal not treated. The red horizontal and blue vertical lines helps in image positioning (red arrow—periapical lesion). (**C**) Endodontic material pushed through the teeth apex onto the maxillary sinus. (**D**) Undiagnosed teeth with non-vital pulp and no typical periapical lesions and slight increased diameter in the periodontal space. (**E**) Endo-perio or perio-endo syndromes related inflammation causing a secondary maxillary sinusitis with non-vital teeth pulp. The red horizontal and blue vertical lines help in image positioning (yellow-arrow loss of alveolar bone and granular tissue formation). (**F**) Alveolar inflammation after teeth removal, green-arrow—(ASD: *alveolitis sicca dolorosa*). Legends: red, blue, and green lines are the orientation of CBCT lines.

**Figure 2:**
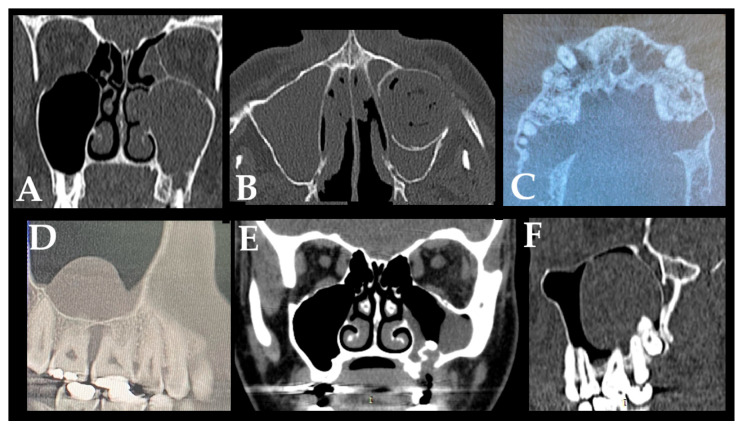
The sources of odontogenic sinusitis (ODS) found in patients’ examination: (**A**) Formation of an odontogenic cyst and secondary maxillary sinusitis. (**B**) A big odontogenic cyst spreading towards the maxillary sinus and causing a visible sinus anterior wall deformity and sinusitis. (**C**) Multiple odontogenic infections, such as periapical lesions, cysts, endo-perio and perio-endo presence in one single patient. The CBCT is less readable because of patient movement during the study. (**D**) Atypical secondary sinus infection caused by poor periodontal status with vital tooth. (**E**) Not diagnosed and not properly closed oro-antral communication after a tooth removal. A classic CT performed between a combined surgical and laryngological intervention (FESS and oro-antral fistula closure). (**F**) Unerupted teeth in the maxillary antrum causing a cyst formation or a secondary mucous accumulation and possible inflammation formation. This cyst leads to ODS due to its large size, disturbed oro-antral flow and drainage, and causes ODS symptoms.

**Figure 3:**
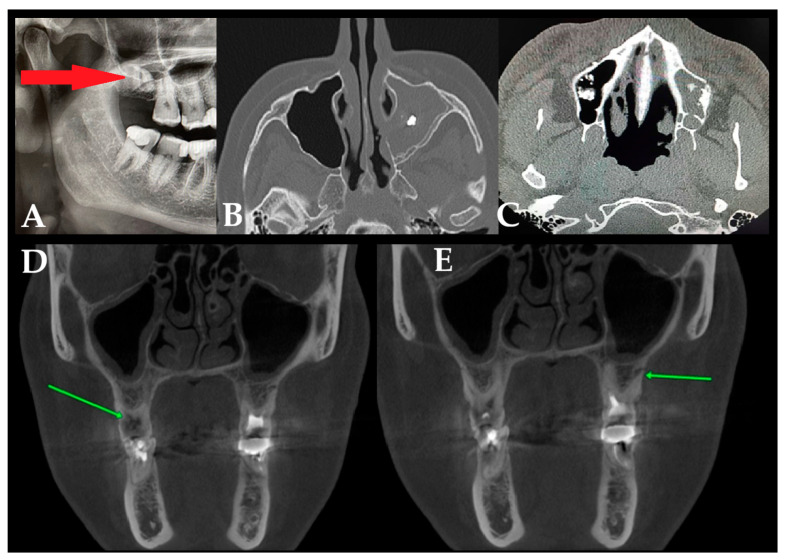
The sources of odontogenic sinusitis (ODS) found in patients’ examination: (**A**) Retained root associated with maxillary sinusitis, demonstrated on panoramic reconstruction/OPG (red arrow). (**B**) Endodontic material used for dental root treatment (CBCT axial view). (**C**) Bone filling material used for socket preservation, bone fillings, sinus lift materials, and other materials that protruded towards the maxillary sinus, like xenograft, allograft or autograft bone materials. In the presented case, the right maxillary sinus was without any changes after the sinus lift procedure. Left maxillary sinus without ventilation with xenograft bone spread within the sinus that caused an ODS. (**D**,**E**)—Unfinished and long-forgotten dental treatment: the left arrow indicates a dental crown destroyed by caries, while the right arrow indicates unfinished endodontic treatment (green arrows).

Based on the author’s findings and the literature [1,2,3,4,5,6,7,8,9,10,11,12,13,14,15,16,17,18,19,20,21,22,23,24,25,26,27,28,29], the most common causes of ODS might be as follows—(a–o) etiologically based on the author’s observational study:

(a) Endodontic disease (dental pulp necrosis, granuloma/cyst formation, apical periodontitis, unfinished endodontic treatment);
(b) The combined endo-perio, perio-endo-inflammations, related to both periodontal inflammation and endodontal status of each tooth;
(c) Periapical inflammation, granuloma, and cyst formation in the adjacent root, periapical complications;
(d) Undiagnosed and complicated dental pulp necrosis;
(e) Periodontal disease and marginal periodontitis, poor periodontal status;
(f) Oro-antral communication, OAC;
(g) Oro-antral fistula, OAF;
(h) Retained root, teeth—with or without cyst formation;
(i) Foreign dental materials: zinc-oxid, gutta-percha, xenograft bone (or other bone), dental fillings, and bone used as regenerative materials;
(j) Sinus lift procedures, dental implant placement, and regenerative techniques used for alveolar bone (bone grafting);
(k) Endodontic material inside the maxillary sinus (differential diagnosis with aspergilloma, fungal infection, and occurrence of both conditions simultaneously);
(l) Unfinished or undiagnosed dental treatment (>6 months after past treatment);
(m) Other possible etiological factors related to teeth and/or dental treatment procedures and the scope of dentoalveolar treatments;
(n) Bone inflammation, alveolitis, maxillary bone osteomyelitis (ex., tooth-related BRONJI/MRONJI (Bisphosphonate-related osteonecrosis of the jaws/Medicament-related osteonecrosis of the jaws) with primary or secondary relation to teeth/dental status;
(o) Other unusual, rare, and atypical possible syndromes, diseases, and conditions related to teeth and tooth-bearing structures.

## 3. Results

### 3.1. Cohort Characteristics

Of the 120 radiological records initially reviewed, 85 met the inclusion criteria and were included in the final analysis. The study group comprised 53 women (62.4%) and 32 men (37.6%). The median age of the overall cohort was 40 years; the median age was 43 years in men and 37 years in women, without a statistically significant difference between the groups (*p* = 0.44). This indicates that both groups are comparable in terms of age.

Because the study was restricted to cases with radiological findings consistent with odontogenic sinusitis, ODS represented the core diagnosis in the entire included cohort. Any prior procedures, postoperative states, or concomitant conditions were recorded separately and should not be interpreted as alternative primary diagnoses.

Table 2 describes the patients (32 men and 53 women) in terms of demographics and clinical diagnoses. Gender did not significantly differentiate the prevalence of any of the diseases mentioned (all *p*-values > 0.05). Acute Odontogenic sinusitis is by far the most common diagnosis, occurring in 82.4% of all study participants. The prevalence in men (81.3%) and women (83.0%) is almost identical (*p* = 1.00). Sinus mucocele occurred in 42.4% of patients. Although it was reported more frequently in men (50.0%) than in women (37.7%), this difference did not reach significance (*p* = 0.37). Radicular cyst occurs with similar frequency in both sexes (total 42.4%, *p* = 0.65). In the case of rarer diseases, no gender dependence was found, although in some cases, certain numerical trends can be seen:

- Silent sinus syndrome occurred more frequently in men (15.6% vs. 3.8% in women), but with this sample size, the result remains statistically insignificant (*p* = 0.10).
- Foreign bodies were noted in 9.4% of patients, with a similar distribution in both sexes (*p* = 0.47).
- Ossifying fibroma occurred exclusively in women (5.7%) in the analyzed group, but due to the small number of cases (*n* = 3), the result is not statistically significant (*p* = 0.29).

The results suggest that anatomical or dental factors are more important than patient gender and age in the maxillary sinus pathology included in this study.

Status after Le Fort I osteotomy is the only feature in this comparison that shows a statistically significant difference between the sexes (*p* = 0.03). Men were significantly more likely to have a history of Le Fort I surgery (usually associated with the treatment of malocclusions or trauma) than women (15.6% vs. 1.9%). The most common additional diagnosis was status post-tooth extraction, occurring in 28.2% of all participants. This frequency was similar in women (30.2%) and men (25.0%), with no statistical difference (*p* = 0.80). The high percentage of patients with tooth extractions (28.2%) and orosinus fistulas (15.3%) confirms that the study group includes patients with problems of a significant dental (odontogenic) origin.

The results confirm that the patients studied primarily had maxillary sinus disease (which correlates with the odontogenic diagnoses in Table 2). The disease occurs with similar frequency on the right and left sides, regardless of gender. The rare involvement of the ethmoid sinus (particularly the posterior one) and turbinates suggests that the disease processes in this group are localized, not diffuse (as is the case with sinus polyposis or chronic allergic rhinitis) (Table 3).

Both the need for root canal treatment and the presence of periapical lesions were distributed across the study population, regardless of patient gender (Table 4). The data confirm that the study group largely consists of patients with stomatognathic problems, nearly two-thirds of the group had undergone root canal treatment and demonstrated pathological lesions. Etiological analysis revealed no sexual dimorphism; men and women were similarly affected by conditions caused by specific teeth or anatomical defects. The most important clinical finding from the gathered study suggests that in over a third of patients (35.3%), the cause of problems is the first upper molar, making it a key point in the differential diagnosis.

Table 5 presents the structure of diagnostic method utilization in the 85-patient group. The results indicate a very high degree of utilization of advanced imaging techniques while maintaining traditional screening methods. The most frequently used examination in the entire group was CBCT (95.3%). This high percentage (almost every patient) indicates that CBCT has become the standard in the diagnosis of odontogenic sinus diseases. The lack of statistical significance (*p* = 0.63) indicates that this examination is ordered equally often in women and men. An interesting correlation was noted in the case of panoramic X-ray (orthopantomogram). Although a total of 85.9% of the study participants underwent this procedure, a gender disparity was evident: Women underwent this procedure significantly more often (92.5%) than men (75.0%). The result of *p* = 0.049 is exactly at the borderline of statistical significance. Waters X-ray (92.9%) is the second most common examination after CBCT. This high frequency is likely due to its standard radiological examination in outpatient ENT diagnostics. No gender differences were observed (*p* = 0.19). MRI (1.2%) was performed in only one patient (a woman). This confirms that MRI in the diagnosis of odontogenic sinusitis is not a routine examination, but rather a targeted one (e.g., when fungal or neoplastic lesions are suspected).

### 3.2. Dental Aspects of Odontogenic Sinusitis in CBCT

**Figure 4:**
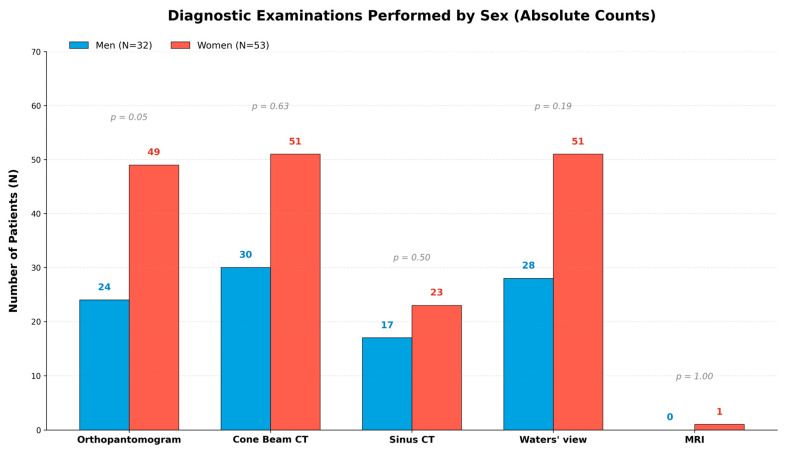
Diagnostic imaging examinations performed in the study group are shown overall and by sex. Data are presented for the total cohort (*N* = 85), men (*N* = 32), and women (*N* = 53), with *p* values for sex-based comparisons.

**Figure 5:**
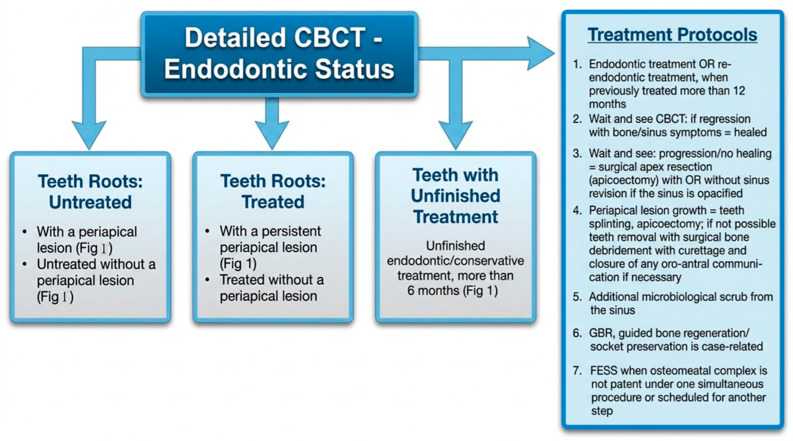
Proposed protocol based on CBCT images on endodontic-related factors influencing ODS.

**Figure 6:**
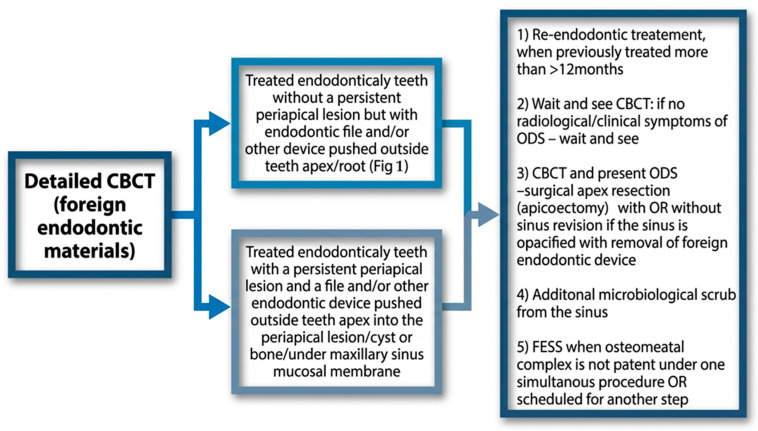
Proposed protocol based on CBCT images on endodontic treatment/complications-related factors influencing ODS.

**Figure 7:**
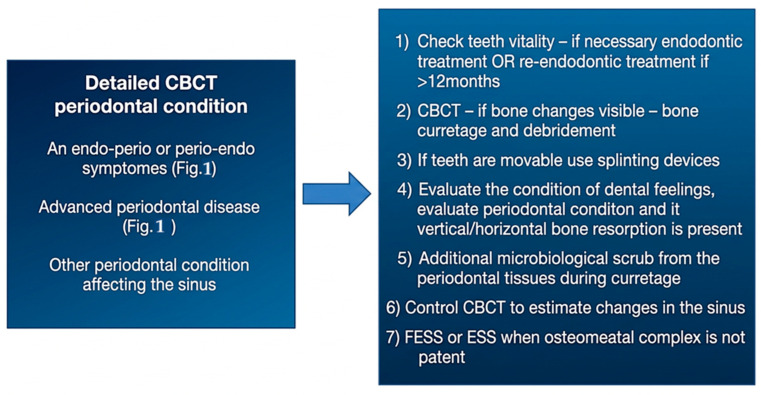
Proposed protocol based on CBCT images on periodontal tissue status/condition-related factors influencing ODS.

**Figure 8:**
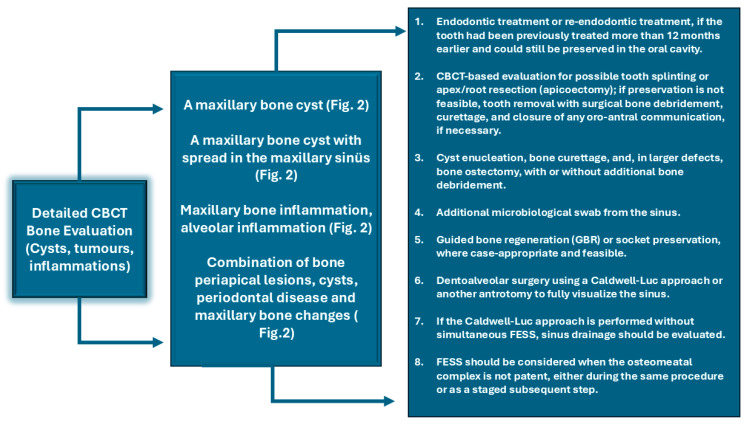
Proposed protocol based on CBCT images on maxillary bone and sinus bone condition-related factors influencing ODS.

A great majority of factors can influence the condition of the surrounding bone and sinus structures. It is worth noting that the condition of each tooth, together with the status of the adjacent periodontal tissues and surrounding bone, may substantially influence the development of ODS. In the authors’ retrospective study on CBCT, the following classifications of ODS occurrence were presented based on the sixteen mostly found in the authors’ database (Figure 1, Figure 2 and Figure 3). The most common ones were related to endodontic treatment and its modalities. Secondly, the incidence of periodontal and/or periodontal-endodontic and co-existing factors also played a role in ODS occurrence. Lastly, the scope of each dento-alveolar surgery and the occurrence of each iatrogenic condition, such as OAC and OAF, were also important factors in ODS. In rare cases, a combination of all situations might be present in one single patient, and because of the following, a necessary team approach is mandatory. The authors indicate that the understanding of each symptom of ODS found in CBCT can greatly improve patients’ outcomes and results from scheduled treatment plans. Each radiological situation in CBCT was responsible for ODS occurrence in different stages, intensification (Figure 1, Figure 2 and Figure 3), and the necessity for one of the following proposed algorithms (Figure 4, Figure 5, Figure 6, Figure 7 and Figure 8).

### 3.3. Author’s Proposal

**Figure 9:**
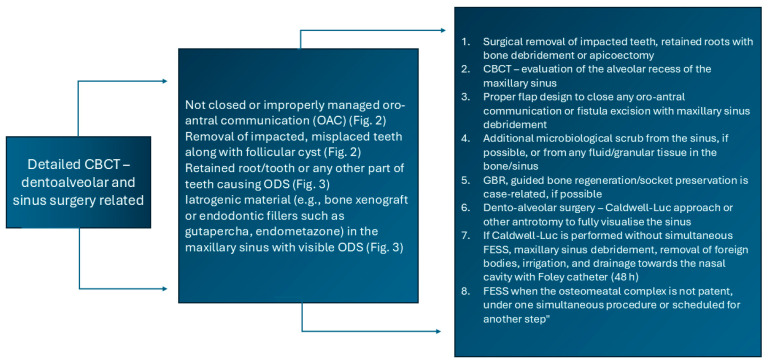
Proposed protocol based on CBCT images on dento-alveolar or sinus-related procedures influencing ODS.

Based on the CBCT findings observed in the study group, the authors organized the most frequent etiologic patterns of ODS into a 16-category classification and presented a corresponding imaging-guided management proposal (Table 1; Figure 4, Figure 5, Figure 6, Figure 7, Figure 8 and Figure 9). The proposed framework linked the radiologic appearance of ODS-related factors with the main treatment approaches used in clinical practice.

### 3.4. Used Treatments and Approaches

Analysis of data regarding the therapeutic procedures used in 85 patients indicates a predominance of combined surgical treatment (Table 6). The choice of treatment methods was dictated by the clinical condition, not the patient’s gender, as indicated by the lack of statistical significance in all categories (all *p* values > 0.05). Antrotomy and revision (91.8%) was the most frequently performed procedure, suggesting that almost all patients required direct opening and surgical clearance of the sinus cavity from inflammatory or pathological masses. The high rate of Caldwell-Luc procedures (75.3%, more common in men—81.3% vs. women—71.7%) indicates a significant number of cases requiring a wide surgical approach, for example, to remove foreign bodies or polyps. The frequent removal of cysts (67.1%) and polyps (58.8%) confirms the chronic and hypertrophic nature of inflammatory lesions in the study group. In over half of the patients, treatment included eliminating the dental focus of infection and simultaneously closing the oral–sinus junction. This is a key element in the treatment of odontogenic sinusitis (ODMS). Osteoplasty, which was frequently performed (62.4%), was likely associated with the reconstruction of defects left by removed dental cysts (which occurred in 58.8% of the study participants).

Culture results from 85 patients with maxillary sinusitis showed a wide diversity of pathogen species, which is characteristic of dental infections (polymicrobial flora). As with treatment methods, no statistically significant differences in the prevalence of specific microorganisms were noted between women and men (all *p* values > 0.05) (Table 7). Regardless of the microbiological flora, surgical approach with adequate diagnostics resolved patient problems in each case (*n* = 100%).

The presence of species such as *Streptococcus sanguinis* (4.7%), *Bifidobacterium denticolens* (3.5%), and *Actinomyces odontolyticus* (1.2%) was noted. Their presence in the sinus directly confirms the dental etiology of the inflammation (infiltration of flora from the periodontium or root canals). The presence of *Escherichia coli* (4.7%), *Serratia marcescens* (2.4%), and species of the genera Enterobacter and Klebsiella is noteworthy. The appearance of these Gram-negative and intestinal bacteria in the sinuses often indicates superinfection in chronic conditions or weakened mucosal defense mechanisms. The presence of anaerobic species such as *Prevotella buccae*, *Veillonella dispar*, and *Finegoldia magna* is typical of purulent inflammatory processes occurring in oxygen-limited conditions, as occurs in a blocked maxillary sinus. In the study group, several patients were identified with the fungal pathogens *Candida species* and *Candida albicans* (a total 4.7%). These are the most common opportunistic fungi found in the oral cavity and sinuses. The microbiological picture in the study group is nonspecific and highly dispersed, with no single dominant pathogen. The predominance of oral bacteria and the presence of fungi support the hypothesis of a complex, often iatrogenic (odontogenic) cause of the disease. The lack of correlation between gender and pathogen type suggests that the sinus microbiota in disease processes depends more on local anatomical conditions and the source of infection than on gender-related systemic factors. Based on the data, no statistically significant relationship can be established between the type of procedure and the need for CBCT [39,40,41,42,43,44,45,46,47,48,49]. Although the Caldwell-Luc procedure predominates in the group of patients who underwent CBCT, due to the small number of patients without CBCT (*N* = 4), these differences do not reach statistical significance. The study is insufficiently powered to clearly link the type of procedure with the need for CBCT, which is also confirmed in other studies [49,50,51,52,53,54,55,56,57,58,59] (Table 8).

A statistically significant association was found between the FESS procedure and the performance of a CT scan of the sinuses (*p* = 0.04).

## 4. Discussion

The main finding of this retrospective CBCT-based series is that odontogenic sinusitis was associated with a broad range of dentoalveolar etiologies rather than a single dominant pattern. In our material, the most relevant findings were related to endodontic/periapical disease, periodontal or combined endo-periodontal pathology, post-extraction inflammatory changes, oro-antral communication/fistula, retained dental structures, odontogenic cystic lesions, and treatment-related foreign materials or graft-related complications. Our results, therefore, support the concept that ODS should be approached as a heterogeneous disease process in which careful analysis of the maxillary dentition, alveolar bone, and sinus floor relationship is essential for identifying the infectious source and planning treatment [5,6,7,8,9,10,11,12,13,14,15,16].

Maxillary sinusitis can result from various etiological factors, including inflammatory, iatrogenic, traumatic, and cystic or tumorous causes, among others; however, rhinogenic and odontogenic origins are the most common. The spectrum of bacterial involvement and the clinical presentation may be quite similar across different forms of sinusitis; however, in each case, detailed clinical and radiological evaluation should be complemented by additional microbiological swabs [9,10,11,12,13,14]. Odontogenic sinusitis (ODS) most commonly manifests as chronic unilateral maxillary sinusitis, although bilateral involvement of both maxillary sinuses is also possible. In most cases, symptoms persist for longer than three months [14,15,16]. Its occurrence and frequency are strongly influenced by dental status, oral hygiene, and previous surgical procedures in the oral cavity, and its effects on the maxillary sinus may be difficult to assess. ODS is frequently misdiagnosed, undertreated, and insufficiently recognized by otolaryngologists [1,2,3,4,5]. According to Psillas et al., approximately 30% of cases of unilateral maxillary sinusitis have a dental etiology, and many of these cases remain undiagnosed [6]. On the other hand, the study by Nurchis et al. concluded that approximately 55.97% of ODS cases were caused by iatrogenic factors, whereas the first and second molars (35.6% vs. 22%, respectively) were the most commonly involved teeth [9]. Although molars appear to be the most frequent etiological factor, in the authors’ opinion, any tooth in close proximity to the maxillary sinus may contribute to the development of ODS, as is also supported by the present retrospective CBCT study. Furthermore, the authors also conclude that the first molar-related ODS is quite common, regardless of its endodontic, periodontic, or surgery-related condition, which can be seen in the authors’ findings (Figure 1, Figure 2, Figure 3, Figure 4, Figure 5, Figure 6, Figure 7, Figure 8 and Figure 9). In our cohort, orthopantomography was more frequently available in women than in men. Although this was not a primary study endpoint, it may reflect differences in referral pathways or a greater likelihood of men and women presenting with prior routine dental imaging [8,9,10,11,12,13,14,15,16,17,24,25,26,27,28,29,30,31,32,33,34,35,36,37,38,39,40,41,42,43,44].

It seems that the condition of teeth, tooth-bearing structures, and surrounding structures in the oral cavity influences the maxillary sinus condition and occurrence of ODS, in the same way as any surgical/dental procedures made and their possible iatrogenic impact on the maxillary sinus [1,6,7,8,14,15,16,17,18,19,20,21]. Many undiagnosed odontogenic factors lead to ODS. In the presented Author’s retrospective study, we estimated the most common etiological factors causing ODS and divided them into (A–P) etiological factors, and possible treatment algorithms based on Figure 1, Figure 2, Figure 3, Figure 4, Figure 5, Figure 6, Figure 7, Figure 8 and Figure 9 were proposed. The practical value of this classification lies in organizing the most frequently observed radiological patterns into an imaging-oriented framework that may facilitate communication between dental and otolaryngological teams. It should therefore be interpreted primarily as a clinical and radiological aid derived from retrospectively observed cases, rather than as a formally validated diagnostic system. The infection spread in the maxillary sinus is greatly related to the condition of the teeth and the degree of possible inflammation, cystic formation, the quality of periodontal tissues, and many other related factors [28,29,30,31,32,33,34,35,36,37,38,39,40,41,42,43,44]. It seems that the symptomatology of sinusitis remains the same, while the possible etiological factors of ODS are most often confirmed in a detailed CBCT evaluation [1,7,8,9,10,21,22,23,24,25,26]. Quite often, when RHS/maxillary sinusitis is not responding well to any treatment, possible odontogenic-related factors should be taken into consideration [5,6,7,8,9,10,11,12,22,23,24,25,26,27]. That is why, because of many similarities in sinus symptoms, the study authors focused mostly on CBCT-related symptoms, confirming the presence of ODS at various stages of local advancement and the scope of possible noticed symptoms [36,37,38,39,40,41,42,43,44].

The presented causes of sixteen different etiological factors causing odontogenic sinusitis represent the most commonly found factors in the author’s retrospective studies (Table 1). So far, studies briefly describing etiological factors of ODS in CBCT are missing. The authors presented sixteen different situations that can not only be fully evaluated in CBCT but can also be easily scheduled for corresponding dental, surgical, or laryngological procedures according to their radiological manifestation. Based on the following CBCT images, it is possible to describe the most suitable direction for combined dental-surgical approaches [1,12,13,14,15,16,17,18,19,20,21,22,23,24,25,26,27,28,44,45,46,47,48,49,50,51,52]. The authors would want to distinguish herein different situations related to the etiological factors of ODS that had been differentiated between those related to endodontic treatment, complicated endodontic treatment, periodontal condition-related, bone condition, and those related to dentoalveolar surgery procedures near the maxillary sinus. The following etiological factors of ODS can be treated in quite similar and linked dental, surgical, and laryngological approaches. Teeth endodontic or re-endodontic treatment followed by apicoectomy or related procedures seems to be the most common once made [1,16,17,18,19,20,21]. Secondly, periodontal debridement and curettage with endodontic treatment can also reduce ODS. Teeth splinting is reserved for larger periapical cysts and tumors that are related to ODS. In any case of teeth removal, a detailed examination of the communication between the maxillary sinus is necessary, and then an oro-antral communication closure is mandatory (Figure 3). When teeth or dental roots are the source of inflammation and ODS, they should be removed, and when possible, any accurate dento-alveolar grafting procedure could be considered [1,2,3,4,5,6,7,8,9,10,11,12]. Larger cysts and hypoventilated maxillary sinuses should be considered for either the endoscopic FESS/ESS approach or an open surgery like the classic Caldwell-Luc antrotomy approach. Iatrogenic situations such as dental materials extrusion towards the sinus, formation of aspergilloma/fungus ball based on zinc oxide-eugenol fillings from endodontic treatment, or removal of any other foreign body should be considered for FESS/ESS or an open surgery with antrostomy or a Caldwell-Luc’s-like procedure [27,28,29,30,31,32,33,34,35,36,37,38,39,40,41,42,43,44,45,46,47,48,49,50,51,52,53,54,55,56,57,58,59]. Regardless of whether there is a periapical lesion, cyst, or bone inflammation/osteomyelitis, each time a bone debridement is performed, followed by microbiological scrub and/or curettage, an ostectomy or other procedure should be used to minimize its negative effect on the maxillary sinuses [1,14,15,16,17,18,19,20,21,22,23,24,25,26,32,33,34]. CBCT should be considered as the diagnostic tool of choice, followed by clinical oral cavity examination and teeth testing for cold stimulus. The author’s proposal is presented in Figure 4, Figure 5, Figure 6, Figure 7 and Figure 8.

Most commonly, ODS is treated by approaches known from dental surgery, combined with endodontic and/or conservative and periodontal treatment. When the scope of ODS exceeds the maxillary sinus, causing stomatal complex patency and other related factors, endoscopic sinus surgery (ESS) is also mandatory to improve sinus ventilation and healing, and decrease related sinus symptoms [8,9,10,11,12,25,26,27,28,29,30,31,32,33,34,35,36,37,38,39,40,41,42,43,44,50,51,52,53,54,55,56,57,58,59]. It is worth noting that the indication for a FESS/ESS approach can be readily evaluated on the basis of CBCT assessment of the maxillary sinus, allowing the procedure to be planned either as a simultaneous intervention or as a standalone treatment [6,7,8,9,10,11,12,13,22,23,24,25,26,27,28,29,30]. In our cohort, sinus CT appeared to be more commonly obtained in patients who underwent FESS, which may reflect preoperative imaging preferences when endoscopic treatment is being considered. However, this observation should be interpreted cautiously, given the retrospective design and the limited subgroup size. From an imaging perspective, our findings support a complementary rather than exclusive role for CBCT. In the present series, CBCT was particularly valuable for identifying dentoalveolar pathology, including periapical lesions, periodontal bone loss, retained roots, foreign materials, and the spatial relationship between dental disease and the maxillary sinus floor [26,27,28,29,30,31,32,33,34,35,36,37,38,39,40,41,42,43,44,45,46,47,48,49,50,51,52,53]. However, these findings should not be interpreted as meaning that CBCT alone is always sufficient in all patients with suspected ODS. Sinus CT remains important when the full extent of sinonasal disease, osteomeatal complex obstruction, adjacent sinus involvement, or extra-sinus spread must be assessed [4,21,44,45,46,47,48]. In the authors’ opinion, each case should be assessed individually, with particular consideration given to osteomeatal complex patency, the degree of sinus ventilation, the indication for open sinus surgery, and the type of intra-sinus pathology present, including cystic lesions, polyps, mucous retention cysts, foreign bodies, aspergilloma, or other conditions [1,19,20,21,22,23,24,25,44,45,46,47,48,49,50,51,52]. It is worth pointing out that in each case, a detailed team approach is mandatory. Many authors concluded that dentists, surgeons, and otolaryngologists should be working in collaboration all together to prevent any potential sinusitis recurrences and improve patients’ overall sinus quality [2,5,9,11,12,13,14,15]. The study authors emphasize that a team effort in the treatment of ODS is a very important aspect, similar to performing a detailed CBCT jaw bone and sinus evaluation.

Many known studies and papers on ODS describe different algorithms in treatment, diagnostics, and possible protocols that could be used for ODS [5,6,7,8,9,10,11,12,13,14,15,16,17,18]. Quite often, the role of a general dentist in ODS diagnostics and treatment is underestimated. That is why, from the study authors’ point of view, CBCT diagnostics is not only important in ODS confirmation but also requires a great collaboration with a general dentist on every step of the treatment. The role of CBCT in influencing endodontic treatment and its accuracy is very important [14,15,24,28,30,31,32,33,34,36,37,38,39,40,41,42,43,44,59]. Mostly an endodontic and/or re-endodontic treatment as a sole procedure is sufficient; however, in some cases, additional apicoectomy or other dento-alveolar surgery should be scheduled to fully remove the cause of ODS. This combined approach is very important to establish and limit the spread of ODS, prognosis, and its degree. Many authors, such as Nurchis et al., indicated that improperly treated infection might spread to other sinuses, and adjacent anatomical areas, and greatly influence patients’ quality of life (QOL) as well as the occurrence of troublesome ODS-related symptoms [9]. The impact on patients’ sinus and bone conditions is also related to the possible treatment modalities that can be used. Treatment modalities of ODS can be different in each case, depending on the condition of the teeth, and bone and periodontal tissue involvement. CBCT helps in treatment planning. Aukštakalnis et al.’s review of 2886 articles included a total of 25 papers for the review and indicated that the Caldwell-Luc approach is limited to selected cases, especially in the removal of foreign bodies, while the endoscopic sinus surgery remains the most common and used nowadays [10,28,33,38,39,40,41,42,43,44]. From the author’s point of view, it is worth understanding that despite the Caldwell-Luc approach being granted to special cases, it is still used nowadays. Any other antroscopic procedure granted by any dento-alveolar approach is equally sufficient to remove any potential ODS factors from the periapical and alveolar recess of the maxillary sinus areas.

The indication for any open or endoscopic surgery is greatly case-related [12,13,14,15,16,17,18,23,24,25,26,27,28,29,30,31,32,33,34,35,36,37,38,39,40,41,42,43,44,45,46,47,48,49]. Many authors advise a combined approach to achieve more satisfactory results. Similar conclusions were made by Craig et al. based on sixteen included papers for the study, which concluded that the ESS approach was not a gold standard alone, because a combined multi-specialized collaboration should estimate the scope of surgical, dental, and laryngological approaches [1,12,14,15,16,17,18,19,20,21,22,23,24,25,26,27,28,44]. It is worth remembering that each approach has its benefits, risks, and limitations, and that in some cases, one surgery is not sufficient to fully help a patient suffering from ODS. Similar conclusions can be drawn from the following paper: when a general dentist helps in ODS treatment, especially in endodontic and periodontal treatment, it is very important. Sabatino et al. study evaluated if a nasal, oral, or combined nasal–oral approach is the most effective one to treat ODS and concluded that all are sufficient; however, the co-occurrence of deviated septum, concha bullosa, enlarged conchae, osteo-metal complex patency, or other similar anatomical situations might influence the treatment model [1,13,21,22,46].

Dental treatment alone is mostly a basis for other surgical, laryngological, or combined treatment approaches. The role of endodontic treatment is very important [14,21,32] in ODS treatment. For endodontic evaluations, cold pulp testing and cone-beam CT imaging are most optimal for confirming pulpal and periapical disease. The role of general dentists, endodontists, and periodontologists should be especially remembered since many cases of periapical odontogenic lesions are underrated and not taken into account [14,15,16,17,33,34,35,36,37,38,39,54].

While RHS syndromes are unilateral, a possible ODS should be taken into consideration [12,13,14,15,16,17,33,34,35,36,37,38,39]. It is also worth noticing that some ODS can be seen bilaterally, especially as presented by the authors in Figure 9. When bilaterally in jaw bones, there are many odontogenic infection sites such as periapical lesions, endo-perio/perio-endo, odontogenic cysts, etc. This finding should also be remembered by clinicians, not only to focus on one-sided RHS, but also to establish the full scope of dental and teeth status to confirm or deny any possible tooth-related sinusitis. Possible misdiagnosed odontogenic sinusitis cases have been briefly reported by many authors so far [1,15,16,17,18,21,22,23,24,25,26,29,32,37]. On the other hand, complicated dental extractions, the occurrence of oro-antral communication (OAC), fistulas (OAF), and the occurrence of iatrogenic sinus infection after alveolar bone augmentation, sinus lift, or other dental procedures can also be potential sources of ODS. Lee et al. evaluated that in the studied material, ten patients (37%) had dental implant-related complications, while eight (29.6%) had dental extraction-related complications, which was also a source of ODS [1,19,20,21,22,23,24,30,31,32,33,34,35,36,37,38,39,40,41,42,43]. It is also worth distinguishing those types of dental and surgical complications that can affect maxillary sinus conditions. Similar features were reported by the study authors as those in Figure 4, Figure 5, Figure 6, Figure 7 and Figure 8. It is worth noting that if these complications had been identified and differentiated earlier on CBCT, the likelihood of ODS development might have been reduced. Establishing a definitive diagnosis of ODS remains challenging; therefore, several diagnostic proposals have been introduced over the years, particularly those based on the modified Delphi method [1,20,21,22,23,24,25,26,27,34,35,36,37,38,39,40,41,42,43,44,45,46,47,48,49,50,51,52,53,54,55,56,57]. Study authors partially agree with the following, mostly because a CBCT examination alone is the most precise tool to establish teeth, bone, and periodontal tissue conditions that might affect the maxillary sinus condition, and because of this, these authors study CBCT usage on ODS evaluation. Furthermore, Whyte and Boeddinghaus’s study on CBCT indicates that this is the most efficient method of evaluating the relation between the teeth roots and the floor of the maxillary [21].

While careful dental examination and CBCT evaluation are essential for the diagnosis and management of ODS, it should also be emphasized that the pharmacological, endoscopic, and otolaryngological treatment approaches may be largely similar to those applied in rhinogenic rhinosinusitis (RHS) [5,6,7,8,9,10,12,13,14,15,33,34,35,36,37,38,39]. Because of a great majority of etiological dental factors, related symptoms, and the scope of changes within the maxillary sinus, the adjacent maxillary bone, and tooth-baring structures, the intensity of each treatment is greatly individual. Regardless of the time, scope, and therapeutic protocol, each ODS can cause some devastating changes in the maxillary sinus as well as adjacent structures. When treated improperly, for too long, and without its full scope, it might lead to some local and general complications. Because ODS can be underestimated, its occurrence time might lead to various complications [23,24,25,26,34,37,40,41,42,43,44,45,46,47,48,49]. When not treated, it can lead to complications that manifest in the jaw bones, sinus, orbital socket, peri/intracranial, and adjacent areas. Both children and adults can suffer from those complications; however, in children, those complications can occur faster and more rapidly [1,20,21,22,23,24,25,26,27,50,51,52,53,54,55,56,57,58,59]. It is worth noting that many ODS have iatrogenic origins, and a clear study of sinus floor, especially before and after a sinus lift procedure, is very important. An important aspect of this was presented by Cosola et al., in which authors described radiographic and histomorphologic evaluation of the maxillary bone after crestal mini sinus lift using absorbable collagen [60].

The scope and degree of combined approaches are greatly dependent on the condition of the osteomeatal complex (OMC), sinus ventilation, possible cyst/tumor spread in the maxillary and sinus walls, condition of the nasal cavity (deviated septum, enlarged conchae), the necessity of teeth removal or just its resection (apicoectomy procedure) and/or endodontic treatment [1,20,21,22,23,24,25,26,27,31,32,33,34,35,36,37,38,39,40,41,42,43,44]. Secondly, the presence of periapical lesions or inflammation, cyst formation, post-extraction oroantral communications, and implant-related procedures appears to represent the most important etiological factors that can be readily identified on CBCT; however, of concern is the fact that their incidence appears to be increasing [1,20,21,22,28,29,30,31,32]. Antibiotics, nasal agents, steroids, and other pharmacological agents are used in both cases, and each clinician is experience-based [25,26,27,32,33,34,35,36,37,38,39,40,41,42,43,44]. The following study emphasizes how important CBCT and team collaboration in ODS treatment are [39,40,41,42,43,44,45,46,47,48,49,50,51,52,53,54,55,56,57,58,59,60].

This study has several limitations. First, it is retrospective in design. Second, it represents a selected referral and treatment cohort rather than an unselected population of all patients with maxillary sinus disease, which limits generalizability. Third, imaging protocols and CBCT fields of view were not uniform. Fourth, the proposed classification was not externally validated, and no interobserver agreement analysis was performed. Fifth, long-term follow-up was incomplete in some cases. Finally, because the study was designed primarily as a radiologic and practice-oriented analysis, the relationship between specific radiologic categories and comparative treatment outcomes could not be tested in a standardized way. The scope of nasal-, sinus- and oral-related symptoms and their severity along with symptom severity grading and nasal endoscopy findings at this stage of surgery was impossible to rationally compare and describe, because of many patient-individual factors. Despite these limitations, the study still provides a clinically relevant overview of the spectrum of odontogenic causes that are detectable on CBCT and supports the need for structured, multidisciplinary evaluation in suspected ODS.

## 5. Conclusions

Odontogenic maxillary sinusitis can have various amounts of teeth- and dental-related etiological factors, which can be easily and fully evaluated in the CBCT. A precise evaluation of each radiograph followed by a close collaboration between dentists, surgeons, and otolaryngologists can greatly improve patients’ sinus conditions, disease spread, and overall quality of life. It is quite important to be aware of the majority of dental etiological factors that might be responsible for ODS. Careful CBCT evaluation allows the clinician to predict the type and extent of the treatment approach to be planned for each individual patient. Presented herein is a radiological CBCT evaluation with a detailed presentation of possible treatment algorithms that might improve treatment outcomes.

## Figures and Tables

**Table 2:** Associated clinical conditions, prior procedures, and concomitant findings in the included ODS cohort, by sex.

Additional Diagnosis	Total *N* = 85	Sex		*p*
		Men (*N* = 32)	Women (*N* = 53)	
(*Status post Le Fort I osteotomy*)	6	5	1	**0.03**
	(7.1%)	(15.6%)	(1.9%)	
(*Functional Endoscopic Sinus Surgery*)	10	6	4	0.17
	(11.8%)	(18.8%)	(7.5%)	
(*Status post tooth extraction*)	24	8	16	0.80
	(28.2%)	(25.0%)	(30.2%)	
(*Oroantral fistula*)	13	5	8	1.00
	(15.3%)	(15.6%)	(15.1%)	
(*Oronasal fistula*)	2	0	2	0.52
	(2.4%)	(0.0%)	(3.8%)	
(*Status post cyst removal*)	7	1	6	0.25
	(8.2%)	(3.1%)	(11.3%)	
(*Maxillary sinus hypoplasia*)	7	5	2	0.10
	(8.2%)	(15.6%)	(3.8%)	
(*Sinonasal Papilloma*)	1	0	1	1.00
	(1.2%)	(0.0%)	(1.9%)	
(*Status post maxillary fracture*)	1	1	0	0.38
	(1.2%)	(3.1%)	(0.0%)	
(*Multiple bone cysts*)	9	3	6	1.00
	(10.6%)	(9.4%)	(11.3%)	
(*Impacted tooth*)	5	0	5	0.15
	(5.9%)	(0.0%)	(9.4%)	
(*Maxillary and maxillary sinus fracture*)	2	1	1	1.00
	(2.4%)	(3.1%)	(1.9%)	
(*Status post Inverted Papilloma resection*)	1	0	1	1.00
	(1.2%)	(0.0%)	(1.9%)	
(*Status post abscess*)	5	2	3	1.00
	(5.9%)	(6.3%)	(5.7%)	
(*Dental roots*)	1	0	1	1.00
	(1.2%)	(0.0%)	(1.9%)	
(*Discomfort due to scarring and adhesions*)	1	1	0	0.38
	(1.2%)	(3.1%)	(0.0%)	

**Table 3:** Location of diseases across gender groups and comparison results.

Anatomical Location of the Disease	Total *N* = 85	Sex		*p*
		Men (*N* = 32)	Women (*N* = 53)	
(*Right maxillary sinus*)	44	15	29	0.51
	(51.8%)	(46.9%)	(54.7%)	
(*Left maxillary sinus*)	43	19	24	0.26
	(50.6%)	(59.4%)	(45.3%)	
(*Right anterior ethmoid cells*)	3	1	2	1.00
	(3.5%)	(3.1%)	(3.8%)	
(*Left anterior ethmoid cells*)	7	1	6	0.25
	(8.2%)	(3.1%)	(11.3%)	
(*Right posterior ethmoid cells*)	1	0	1	1.00
	(1.2%)	(0.0%)	(1.9%)	
(*Left posterior ethmoid cells*)	2	0	2	0.52
	(2.4%)	(0.0%)	(3.8%)	
(*Inferior nasal turbinate*)	5	1	4	0.65
	(5.9%)	(3.1%)	(7.5%)	
(*Middle nasal turbinate*)	1	0	1	1.00
	(1.2%)	(0.0%)	(1.9%)	
(*Nasal septum*)	1	0	1	1.00
	(1.2%)	(0.0%)	(1.9%)	

**Table 4:** Endodontic treatment and lesion occurrence among patients by sex.

	Total *N* = 85	Sex		*p*
		Men (*N* = 32)	Women (*N* = 53)	
(*Endodontic treatment*)				0.25
*Yes*	53 (62.4%)	17 (53.1%)	36 (67.9%)	
*No*	32 (37.6%)	15 (46.9%)	17 (32.1%)	
(*Periapical Lesions)*				0.34
*Yes*	57 (67.1%)	19 (59.4%)	38 (71.7%)	
*No*	28 (32.9%)	13 (40.6%)	15 (28.3%)	

**Table 5:** Types of diagnostic examinations performed in the study group by sex.

(*Diagnostic Examinations*)	Total *N* = 85	Sex		*p*
		Men (*N* = 32)	Women (*N* = 53)	
(*Orthopantomogram*)	73	24	49	**0.05**
	(85.9%)	(75.0%)	(92.5%)	
(*Cone Beam Computed Tomography*)	81 (95.3%)	30 (93.8%)	51 (96.2%)	0.63
(*Sinus CT*)	40 (47.1%)	17 (53.1%)	23 (43.4%)	0.50
(*Waters’ view radiograph*)	79 (92.9%)	28 (87.5%)	51 (96.2%)	0.19
(*Magnetic Resonance Imaging*)	1	0	1	1.00
	(1.2%)	(0.0%)	(1.9%)	

**Table 6:** Types of treatment undertaken in the study group by sex.

(*Type of Treatment Undertaken*)	Total *N* = 85	Sex		*p*
		Men (*N* = 32)	Women (*N* = 53)	
(*Tooth extraction*)	42	15	27	0.82
	(49.4%)	(46.9%)	(50.9%)	
(*Cystectomy of an odontogenic cyst*)	50 (58.8%)	19 (59.4%)	31 (58.5%)	1.00
(*Caldwell-Luc operation*)	64 (75.3%)	26 (81.3%)	38 (71.7%)	0.44
(*Functional Endoscopic Sinus Surgery*)	10	6	4	0.17
	(11.8%)	(18.8%)	(7.5%)	
(*Antrotomy and revision*)	78 (91.8%)	30 (93.8%)	48 (90.6%)	0.71
(*Sinus cystectomy*)	57 (67.1%)	25 (78.1%)	32 (60.4%)	0.10
(*Ethmoidectomy*)	8	3	5	1.00
	(9.4%)	(9.4%)	(9.4%)	
(*Maxillary sinus drainage with a Foley catheter*)	51	19	32	1.00
	(60.0%)	(59.4%)	(60.4%)	
(*Sinus-Ject drainage system*)	1	0	1	1.00
	(1.2%)	(0.0%)	(1.9%)	
(*Apicoectomy*)	18 (21.2%)	9 (28.1%)	9 (17.0%)	0.28
(*Osteoplasty*)	53 (62.4%)	17 (53.1%)	36 (67.9%)	0.25
(*Maxillary sinus polypectomy*)	50 (58.8%)	20 (62.5%)	30 (56.6%)	0.65
(*Closure of oroantral fistula*)	51 (60.0%)	20 (62.5%)	31 (58.5%)	0.82
(*Excision of papilloma and coagulation*)	1	0	1	1.00
	(1.2%)	(0.0%)	(1.9%)	
(*Removal of a foreign body*)	7	2	5	0.71
	(8.2%)	(6.3%)	(9.4%)	
(*En bloc resection*)	5	2	3	1.00
	(5.9%)	(6.3%)	(5.7%)	
(*Fracture reduction and stabilization*)	1	0	1	1.00
	(1.2%)	(0.0%)	(1.9%)	
(*Modified Denker’s procedure*)	1	0	1	1.00
	(1.2%)	(0.0%)	(1.9%)	
(*OMC clearance*)	28 (32.9%)	17 (32.1%)	11 (34.4%)	1.00
(*Revision of the nasal cavity*)	3	1	2	1.00
	(3.5%)	(3.1%)	(3.8%)	
(*Scar revision surgery*)	1	1	0	0.38
	(1.2%)	(3.1%)	(0.0%)	

**Table 7:** Microbiological specimen.

Bacteria and Fungi	Total (*N* = 85)	Sex		*p*
		Men (*N* = 32)	Women (*N* = 53)	
*Staphylococcus aureus*	2 (2.4%)	0 (0.0%)	2 (3.8%)	0.52
*Streptococcus oralis*	1 (1.2%)	1 (3.1%)	0 (0.0%)	0.38
*Candida albicans*	1 (1.2%)	1 (3.1%)	0 (0.0%)	0.38
*Serratia marescens*	2 (2.4%)	0 (0.0%)	2 (3.8%)	0.52
*Prevotella buccae*	2 (2.4%)	0 (0.0%)	2 (3.8%)	0.52
*Enterobacter kobei*	2 (2.4%)	1 (3.1%)	1 (1.9%)	1.00
*Escherichia coli*	4 (4.7%)	1 (3.1%)	3 (5.7%)	1.00
*Candida species*	3 (3.5%)	1 (3.1%)	2 (3.8%)	1.00
*Enterobacter hormaeche*	1 (1.2%)	0 (0.0%)	1 (1.9%)	1.00
*Streptoccocus sanguinis*	4 (4.7%)	1 (3.1%)	3 (5.7%)	1.00
*Bifidobacterium species*	1 (1.2%)	0 (0.0%)	1 (1.9%)	1.00
*Aspergillus fumigatus*	2 (2.4%)	0 (0.0%)	2 (3.8%)	0.52
*Pantoea agglomerans*	1 (1.2%)	0 (0.0%)	1 (1.9%)	1.00
*Actinomyces odontoliticus*	1 (1.2%)	1 (3.1%)	0 (0.0%)	0.38
*Enterococcus faecalis*	2 (2.4%)	2 (6.3%)	0 (0.0%)	0.14
*Enterococcus* spp.	1 (1.2%)	1 (3.1%)	0 (0.0%)	0.38
*Vellionella* spp.	2 (2.4%)	0 (0.0%)	2 (3.8%)	0.52
*Pseudomonas aeruginosa*	1 (1.2%)	0 (0.0%)	1 (1.9%)	1.00
*Finegoldia magna*	1 (1.2%)	0 (0.0%)	1 (1.9%)	1.00
*Bifidobacterium denticolens*	3 (3.5%)	0 (0.0%)	3 (5.7%)	0.29
*Veillonella dispar*	2 (2.4%)	2 (6.3%)	0 (0.0%)	0.14
*Klebsiella oxytoca*	1 (1.2%)	1 (3.1%)	0 (0.0%)	0.38

**Table 8:** Correlation between the amount of FESS and Caldwell-Luca procedures and the computed tomography study.

Procedure	Sinus CT		*p*-Value	OR [95% CI]
	Yes *N* = 40	None *N* = 56		
Caldwell-Luca	30 (75.0%)	34 (75.6%)	1.000	0.97 [0.36–2.60]
FESS	8 (20.0%)	2 (4.4%)	**0.040**	**5.38 [1.07–27.0]**

## Data Availability

Availability of supporting data—the datasets used and/or analyzed during the current study are available from the corresponding author on reasonable request.
